# Complete genome sequence of *Streptomyces* strain VNUA116, a potential biocontrol against phytopathogenic fusarium wilt fungus *Fusarium oxysporum* f. sp. *cubense*

**DOI:** 10.1128/MRA.00706-23

**Published:** 2023-11-07

**Authors:** Mai Thi Thanh Nguyen, Son Truong Dinh, Canh Xuan Nguyen

**Affiliations:** 1 Center for Experimental Biology, National Center for Technological Progress, Hanoi, Vietnam; 2 Faculty of Biotechnology, Vietnam National University of Agriculture, Hanoi, Vietnam; 3 Institute of Agrobiology, Vietnam National University of Agriculture, Hanoi, Vietnam; University of Maryland School of Medicine, Baltimore, Maryland, USA

**Keywords:** complete genome, *Streptomyces* sp., biocontrol, *Fusarium oxysporum* f. sp. *cubense*

## Abstract

Here, we present the whole-genome sequence of *Streptomyces* strain VNUA116 was obtained by combining sequencing data from both PacBio RS II and DNBseq platforms. The complete circular genome is 8,306,919 bp with a GC content of 72.49%.

## ANNOUNCEMENT

**Fig 1:**
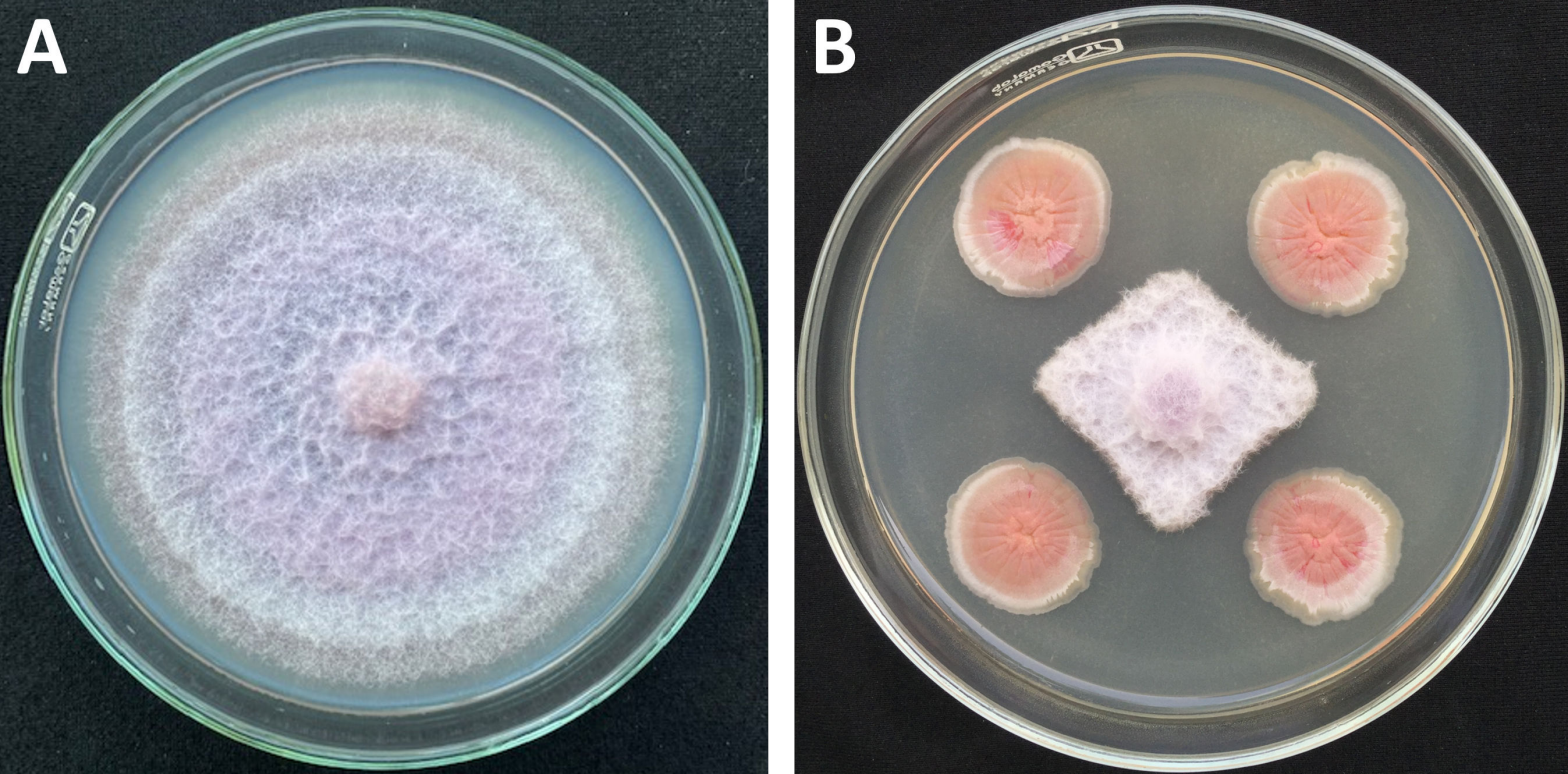
Fungistatic activities of *Streptomyces* strain VNUA116 on the mycelial growth of *Fusarium oxysporum*. (**A**) Growth of *Fusarium oxysporum* f. sp. *cubense* on control medium. (**B**) Inhibition of mycelium growth of *Fusarium oxysporum* by *Streptomyces* strain VNUA116.

The *Streptomyces* VNUA116 strain was isolated from soil collected in a *Fusarium oxysporum*-infected banana plantation in Vietnam, followed by serial dilutions and resulted in a pure culture (1). The strain showed fungistatic activity against banana fusarium wilt fungus (Fig. 1) (2).

A single colony was picked for culture overnight in 30 mL of Gause’s no. 1 liquid medium at 30°C, shaking at 200 rpm for 2 days. Fifteen milliliters of culture broth was used to isolate DNA using a modified DNA isolation procedure [modification: used phenol:chloroform:isoamyl alcohol (25:24:1) before adding chloroform:isoamyl alcohol (24:1) step] (3). The concentration, integrity, and purity of gDNA were determined, and then gDNA was sequenced with the DNBSEQ and PacBio Sequel II platforms at the Beijing Genomics Institute, China.

On the DNBSEQ platform, the single-strand circle DNA library was subjected to BGISEQ-500 sequencer for 150-bp paired-end sequencing according to the manufacturer’s protocol (4). The raw reads were filtered and preprocessed using SOAPnuke v.1.5.5 software (https://github.com/BGI-flexlab/SOAPnuke) (5). The DNBSEQ sequencing obtained 8,764,210 paired-end sequence reads and 157.8× coverage.

On the PacBio platform, the DNA fragments were first treated with Covaris g-TUBE to the appropriate size (about 10–15 kb), then the fragment ends were ligated to form a dumbbell structure using the SMRTbell Template Prep Kit v.1.0 (Pacific Bioscience). The PacBio subreads less than 1 kb long were removed. The reads had N50 = 9,332 bp, N90 = 6,120 bp, max length = 167,462 bp, and mean length = 8.641 bp. Circular-consensus sequences were obtained by comparing subreads to improve the accuracy of single-molecule real-time sequencing. The reads were assembled into unitigs using Canu v.1.5 (estn = 24, npruseGrid = 0, and corOvlMemory = 4) (https://github.com/marbl/canu/releases) (6).

Single-base corrections were then performed using GATK v.1.6–13 (-cluster 2 -window 5 -stand_call_conf 50 -stand_emit_conf 10.0 -dcov 200 MQ0> =4) (https://www.broadinstitute.org/gatk/) to improve the accuracy of the genome sequences. The merging and circularization of the genome was performed by Circlator v.1.5.5 (7). Gene component prediction was performed using Glimmer v.3.02 software (http://www.cbcb.umd.edu/software/glimmer/) with Hidden Markov models (-o * -g * -t * -l linear) (8, 9). For tRNA, rRNA, and small RNA (sRNA) recognition, the RNAmmer v.1.2, tRNAscan-SE v.1.3.1 (–s Species –m Type –gff *. rRNA.gff –f *.rRNA.fq) (10), and the Rfam v.9.1 databases were utilized (–*P* blastn –W 7 –e 1 –v 10000 –b 10000 –m 8 –i subfile –o *.blast.m8) (11).

The *Streptomyces* strain VNUA116 genome contains a single circular chromosome with 8,306,919 bp with a G + C content of 72.49%. The genome was annotated to have 7,535 predicted genes by using 12 databases, including VFDB, ARDB, TREMBL, CAZY, IPR, SWISSPROT, COG, CARD, GO, KEGG, NR, and T3SS. Genome annotation resulted in a total of 71 tRNAs, 21 rRNAs, and 52 sRNAs. Six incomplete prophages were found using the PhiSpy v.3.7.8 (https://github.com/linsalrob/PhiSpy) (default settings) (12). CRISPRCasFinder v.4.2.19 (https://crisprcas.i2bc.paris-saclay.fr/CrisprCasFinder/) (default settings) (13) was used to reveal 11 CRISPR sequences.

## Data Availability

The whole-genome sequence and associated data have been deposited at GenBank (accession number CP130487) and the Sequence Read Archive (SRR25317685 and SRR25317686).
